# Supplementary material for: Development of a personalized digital biomarker of vaccine-associated reactogenicity using wearable sensors and digital twin technology
Source: Commun Med (Lond). 2025 Apr 13;5:115. doi: 10.1038/s43856-025-00840-8 (PMC11994808; doi:10.1038/s43856-025-00840-8)
Supplement: Supplementary file 2 — Supplementary Tables and Figures [file 43856_2025_840_MOESM2_ESM.docx]

### Supplementary Information for:

**Development of a personalized digital biomarker of vaccine-associated reactogenicity using wearable sensors and digital twin technology**

Steven R. Steinhubl, Jadranka Sekaric, Maged Gendy, Huaijian Guo, Matthew P. Ward, Craig J. Goergen, Jennifer L. Anderson, Sarwat Amin, Damen Wilson, Eustache Paramithiotis, Stephan Wegerich

**Supplementary Table 1:**

| **Participant Characteristics (n=88 participants, 104 vaccine doses)** |  |
| --- | --- |
| Mean Age (+SD) | 37.9 (+13.9) |
| Female n (%) | 42 (47.7) |
| Self-Reported Prior COVID Infection n (%) | 11 (10.6) |
| Received an initial vaccine dose n (%)* | 15 (14.4%) |
| Received a second vaccine dose n (%)* | 44 (42.3) |
| Received a third vaccine dose n (%)* | 45 (43.3) |
| Received a dose of Moderna’s mRNA-1273 n (%) of 92 who knew type | 43 (46.7) |
| Received a dose of Pfizer-BioNTech’s BNT162b2 n (%) of 92 who knew type | 48 (52.2) |

#### *Total percentages add up to >100% as 14 participants provided data for 2 doses and 1 person for 3 doses

**Supplementary Figure 1:** Heat maps of the 25 participants with the greatest changes relative to their pre-vaccination baselines in each of 5 individual parameters in order of degree of change from higher to lower. Several participants (numbers 04, 23 and 24) are highlighted as examples of how the relative change in one parameter is not predictive of the change in another.


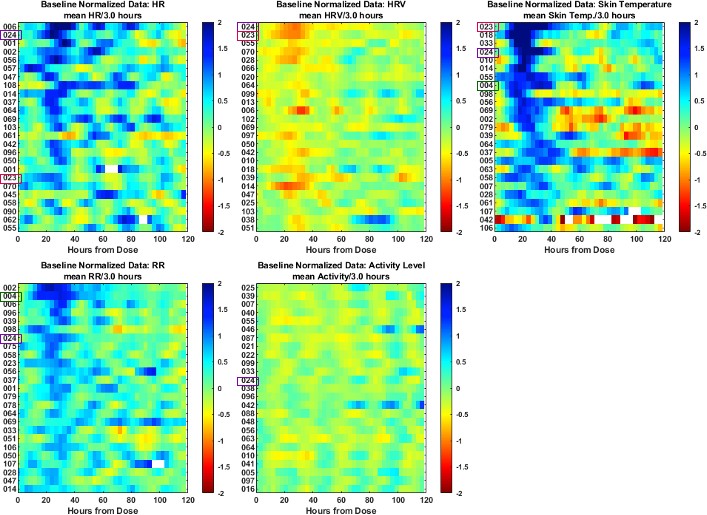


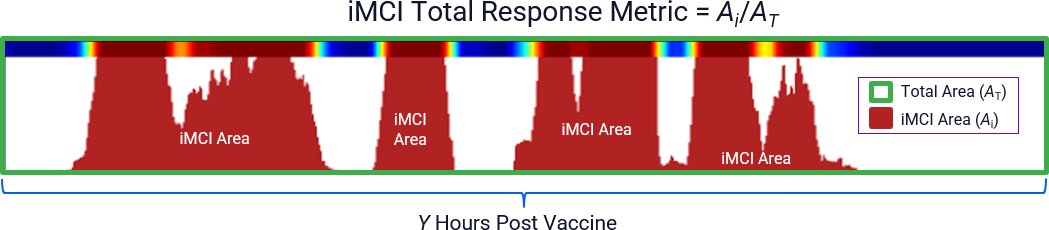
**Supplementary Figure 2:** The MCIR Total Response was defined as A_i_/A_T_ as illustrated in the figure, where A_T_ is the total rectangular area within the time window (the green box) and A_i_ is the area under the curve for MCIR during the window (red area).

**Supplementary Figure 3:** The two top figures (a) and (b) reveal an existence of few outliers. Using the statistical methods such as “median” for anti-spike protein IgG and “generalized extreme studentized deviate test” for T-cell assays we identified outliers and removed them from correlation analyses. There were two outliers in anti-spike protein IgG and three in case of each T-cell assay. One of outliers is common for both T-cell assays and anti-spike protein IgG. This resulted in 17 clinical data points for anti-spike protein IgG and T-cell assays to compare. The resulting correlation between anti-spike protein IgG and T-cell assays in clinical data after outlier removal is shown in two bottom figures (c) and (d) of Supplemental Figure (anti-spike protein IgG and T-cell assay IL-21+: 0.75, one-sided p=0.0003; anti-spike protein IgG and T-cell assay: -0.60, one-sided p=0.007).


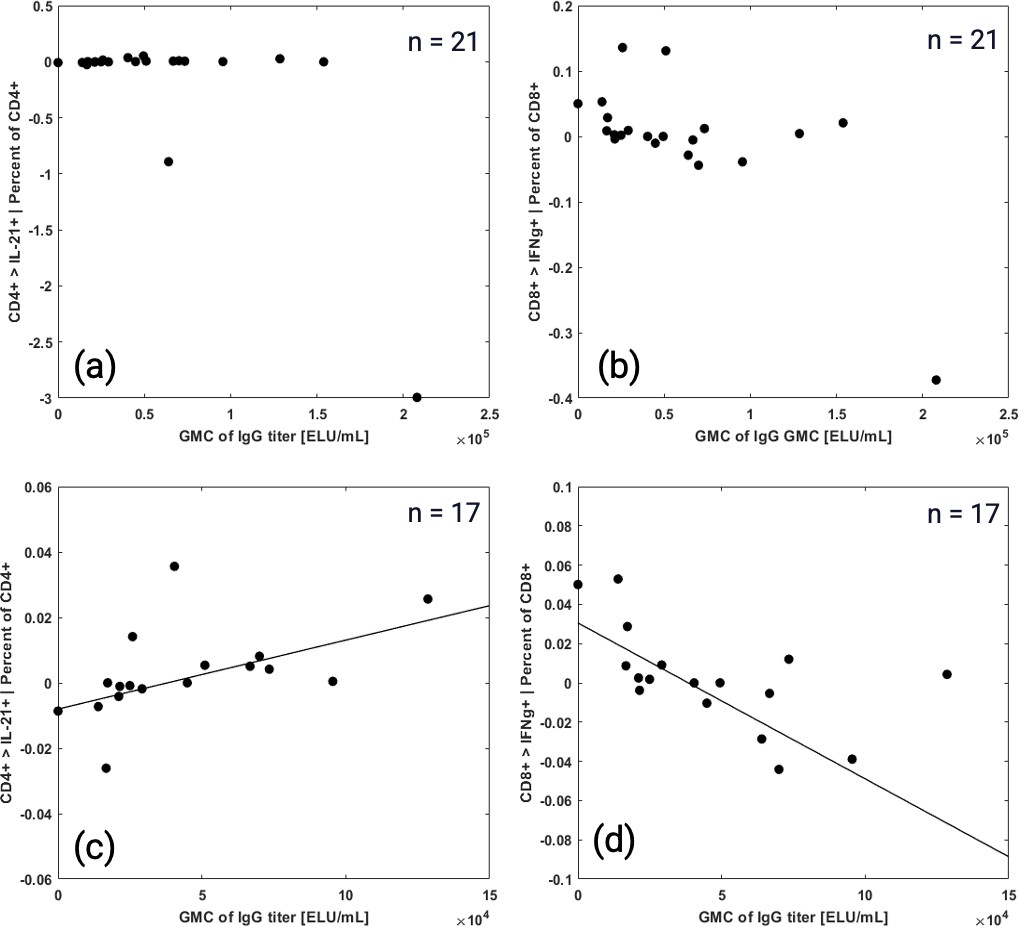


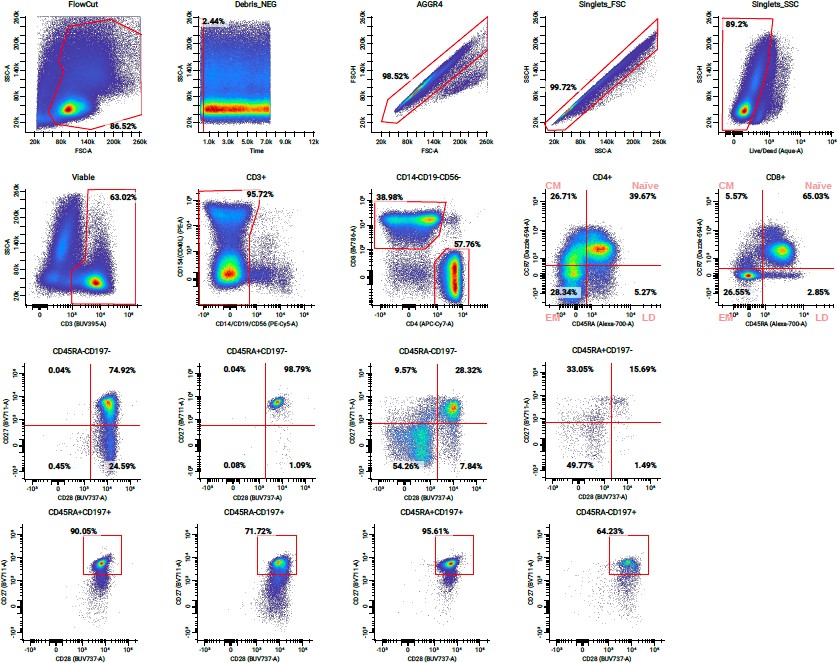
**Supplemental Figure 4:** Multi-parametric flow cytometry gating strategy. Base gates that exclude cellular debris, discriminate between single cells and doublet or higher aggregates, and between live and dead cells were applied to all samples (top row and Viable gate on second row). Cellular subpopulation gates were applied as required. Representative examples of subpopulation gating shown for CD3+, CD4+, CD8+ and the memory subpopulations within the CD4+ and CD8+ populations. EM = effector memory, CM = central memory, LD = Late Differentiated Cells.

C015'(C040l) (l'E-Aj **F Ng(RTC,\j L·2\9" 50-"l** L◄(BV421-A)


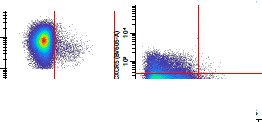


- 1 1 ..59'\

0.1'\

,.. ... ,.. ....

IA (IPU21•

COlflCCCMI

•

,.. ,.. ,.

• • •

... ... ,..

' ™' tf ll'e.,.)

' co1 ,.. fi'f,._l

,.. ,.

##### I I I

i . i§.-

+

§-

# • •


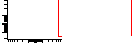


' co1 J'f ,._l

,.. _,_.. ,.


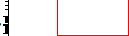

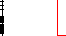


i !

I.

§- i§.-

...

....,.,.....,.. ,..

....,.,......,.. ,..

i§

# •

!

i§.-

IA {81H 1


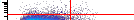


**Q**

*<*""*··.*

\^.^_.,

,.. ...

2 •

- **0 .72'**

ii:

I·

,.. ....

0 .16 \.

C'Olflf CCMI

,..

,.. 2.,-.

Gatedon CD4+, ll •21+

| 25..79'\ | 1.01\. |
| --- | --- |
| 72. 19'\ .. | . ... 1 .0,.1 \. |

•

,.


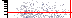
;-

co 1• S(CCRS) 7-A) CXCRS (BV605,>j L· 21 (Alna647-A) Perfodn('PerCP-OfS-6..

# • • •


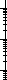

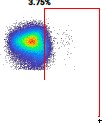


....

COlflCCCMI

•

,.. ,.. ,.

,.. _,_.. ,..

.-

- 10'

C0195"()C:M)tpf,()y1.-.)

'

i .

§-

ClOCM(9,(60S-')

•

....

COlflf CCMI

•

,.. ,.. .,

.-

i .

§-

CIOCM0,,,60S-')

.? -•

i§.-

# •

0.111'

,.. _,_.. ,..

.-

###### ?•

i§.-

.... .... • ,.. ,.. ,..

L41(.A.w...t6.4.?,,()

.... .... ,.. ,.. ,..

•

L41 (Aw.t64?,,()

.?•-

i§

# •


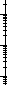

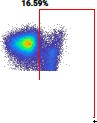


- 1 0'

Pwef olllor-cPq S.s.A)

•

,.. ... ,..

.-

###### ?•

i§.-

- 1 0' ,.. ... ,..

M COlnoa-cPq S.s.A)

•

Gatedon C:04+, CXCRS+ ANDIL-21+

# •

| 1115: sn - | - 3:91 |
| --- | --- |
| ,g ·{i·t· |  |
|  | ,.. _,_.. ,.. |

ii!

•

## I.

##### fl'

COlflfCCflS)fPE

•

**Supplemental Figure 5:** Flow cytometry plots for CD4+ > IL21+ as percent of CD4+ for all participants at all time points. included for both non-stimulated (NS) control and SARS-CoV-2 Spike Protein (S-pool) stimulation. EOS = End of Study (~60 days after last vaccine dose).

**Supplemental Figure 6:** Flow cytometry plots for CD8+ > IFNg+ as percent of CD8+ for all participants at all time points. included for both non-stimulated (NS) control and SARS-CoV-2 Spike Protein (S-pool) stimulation. EOS = End of Study (~60 days after last vaccine dose).

**Supplementary Figure 5:**

NS

S-pool

Baseline D14 post vac1 D14 post vac2 D14 post vac3 EOS Baseline D14 post vac1 D14 post vac2 D14 post vac3 EOS


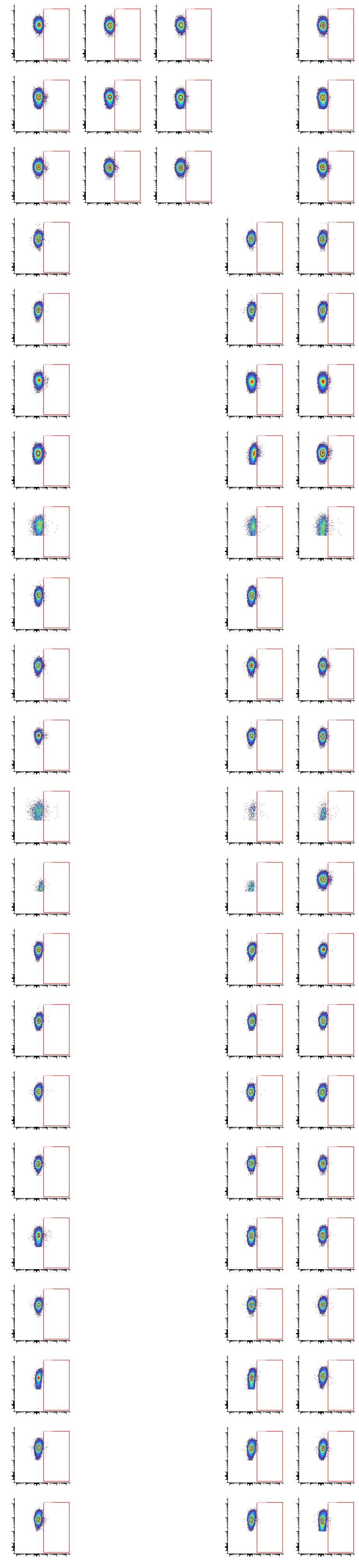


**0.08%**

**0.06%**

**0.07%**

**0.02%**

IL-21 (Alexa 647-A)

IL-21 (Alexa 647-A)

IL-21 (Alexa 647-A)

IL-21 (Alexa 647-A)

**0.22%**

**0.16%**

**0.11%**

**0.06%**

IL-21 (Alexa 647-A)

IL-21 (Alexa 647-A)

IL-21 (Alexa 647-A)

IL-21 (Alexa 647-A)

**0.16%**

**0.12%**

**0.17%**

**0.15%**

IL-21 (Alexa 647-A)

IL-21 (Alexa 647-A)

IL-21 (Alexa 647-A)

IL-21 (Alexa 647-A)

**0.01%**

**0.01%**

**0.01%**

IL-21 (Alexa 647-A)

IL-21 (Alexa 647-A)

IL-21 (Alexa 647-A)

**0%**

**0%**

**0.01%**

IL-21 (Alexa 647-A)

IL-21 (Alexa 647-A)

IL-21 (Alexa 647-A)

**0.29%**

**0.17%**

**0.24%**

IL-21 (Alexa 647-A)

IL-21 (Alexa 647-A)

IL-21 (Alexa 647-A)

**0.11%**

**1.28%**

**0.15%**

IL-21 (Alexa 647-A)

IL-21 (Alexa 647-A)

IL-21 (Alexa 647-A)

**1.18%**

**1.18%**

**1.98%**

IL-21 (Alexa 647-A)

IL-21 (Alexa 647-A)

IL-21 (Alexa 647-A)

**0.04%**

**0.04%**

IL-21 (Alexa 647-A)

IL-21 (Alexa 647-A)

**0.08%**

**0.08%**

**0.1%**

IL-21 (Alexa 647-A)

IL-21 (Alexa 647-A)

IL-21 (Alexa 647-A)

**0.19%**

**0.02%**

**0.03%**

IL-21 (Alexa 647-A)

IL-21 (Alexa 647-A)

IL-21 (Alexa 647-A)

**3.37%**

**3.96%**

**2.56%**

IL-21 (Alexa 647-A)

IL-21 (Alexa 647-A)

IL-21 (Alexa 647-A)

**1.28%**

**0.32%**

**0.35%**

IL-21 (Alexa 647-A)

IL-21 (Alexa 647-A)

IL-21 (Alexa 647-A)

**0.01%**

**0.02%**

**0.02%**

IL-21 (Alexa 647-A)

IL-21 (Alexa 647-A)

IL-21 (Alexa 647-A)

**0.01%**

**0.01%**

**0.01%**

IL-21 (Alexa 647-A)

IL-21 (Alexa 647-A)

IL-21 (Alexa 647-A)

**0.01%**

**0%**

**0.01%**

IL-21 (Alexa 647-A)

IL-21 (Alexa 647-A)

IL-21 (Alexa 647-A)

**0.01%**

**0.02%**

**0.01%**

IL-21 (Alexa 647-A)

IL-21 (Alexa 647-A)

IL-21 (Alexa 647-A)

**0.16%**

**0.02%**

**0.03%**

IL-21 (Alexa 647-A)

IL-21 (Alexa 647-A)

IL-21 (Alexa 647-A)

**0.01%**

**0.02%**

**0.01%**

IL-21 (Alexa 647-A)

IL-21 (Alexa 647-A)

IL-21 (Alexa 647-A)

**0.01%**

**0.02%**

**0.03%**

IL-21 (Alexa 647-A)

IL-21 (Alexa 647-A)

IL-21 (Alexa 647-A)

**0.02%**

**0.02%**

**0.04%**

IL-21 (Alexa 647-A)

IL-21 (Alexa 647-A)

IL-21 (Alexa 647-A)

**0.05%**

**0.02%**

**0.03%**

IL-21 (Alexa 647-A)

IL-21 (Alexa 647-A)

IL-21 (Alexa 647-A)


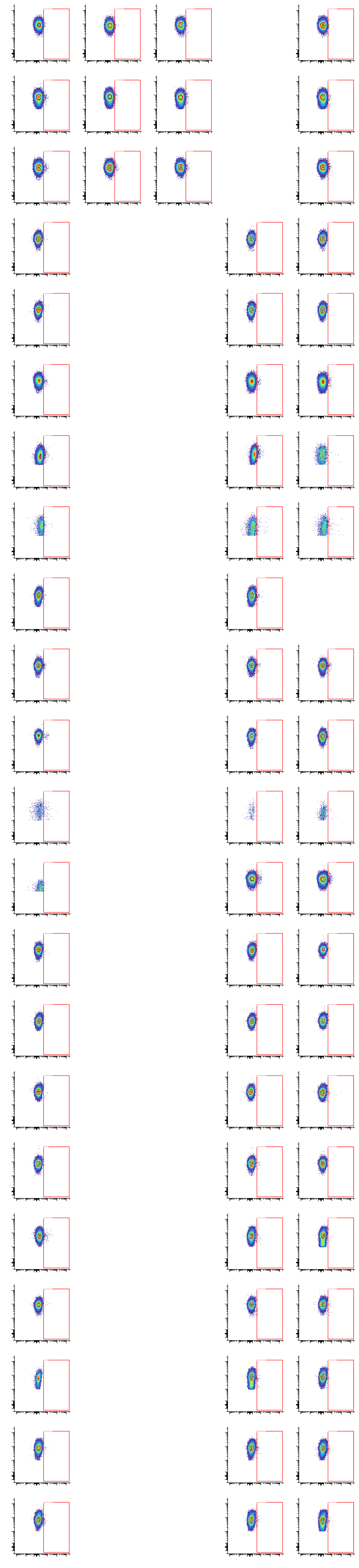


**0.08%**

**0.06%**

**0.07%**

**0.04%**

IL-21 (Alexa 647-A)

IL-21 (Alexa 647-A)

IL-21 (Alexa 647-A)

IL-21 (Alexa 647-A)

**0.16%**

**0.07%**

**0.1%**

**0.08%**

IL-21 (Alexa 647-A)

IL-21 (Alexa 647-A)

IL-21 (Alexa 647-A)

IL-21 (Alexa 647-A)

**0.15%**

**0.11%**

**0.1%**

**0.14%**

IL-21 (Alexa 647-A)

IL-21 (Alexa 647-A)

IL-21 (Alexa 647-A)

IL-21 (Alexa 647-A)

**0.01%**

**0%**

**0%**

IL-21 (Alexa 647-A)

IL-21 (Alexa 647-A)

IL-21 (Alexa 647-A)

**0.01%**

**0%**

**0.01%**

IL-21 (Alexa 647-A)

IL-21 (Alexa 647-A)

IL-21 (Alexa 647-A)

**0.2%**

**0.22%**

**0.19%**

IL-21 (Alexa 647-A)

IL-21 (Alexa 647-A)

IL-21 (Alexa 647-A)

**0.51%**

**1.65%**

**0.4%**

IL-21 (Alexa 647-A)

IL-21 (Alexa 647-A)

IL-21 (Alexa 647-A)

**3.55%**

**2.65%**

**2.75%**

IL-21 (Alexa 647-A)

IL-21 (Alexa 647-A)

IL-21 (Alexa 647-A)

**0.03%**

**0.06%**

IL-21 (Alexa 647-A)

IL-21 (Alexa 647-A)

**0.08%**

**0.12%**

**0.1%**

IL-21 (Alexa 647-A)

IL-21 (Alexa 647-A)

IL-21 (Alexa 647-A)

**0.21%**

**0.04%**

**0.02%**

IL-21 (Alexa 647-A)

IL-21 (Alexa 647-A)

IL-21 (Alexa 647-A)

**6.36%**

**1.47%**

**1.53%**

IL-21 (Alexa 647-A)

IL-21 (Alexa 647-A)

IL-21 (Alexa 647-A)

**0.66%**

**0.32%**

**0.34%**

IL-21 (Alexa 647-A)

IL-21 (Alexa 647-A)

IL-21 (Alexa 647-A)

**0.01%**

**0.02%**

**0.01%**

IL-21 (Alexa 647-A)

IL-21 (Alexa 647-A)

IL-21 (Alexa 647-A)

**0.01%**

**0.02%**

**0.01%**

IL-21 (Alexa 647-A)

IL-21 (Alexa 647-A)

IL-21 (Alexa 647-A)

**0.01%**

**0.01%**

**0.03%**

IL-21 (Alexa 647-A)

IL-21 (Alexa 647-A)

IL-21 (Alexa 647-A)

**0%**

**0.02%**

**0.01%**

IL-21 (Alexa 647-A)

IL-21 (Alexa 647-A)

IL-21 (Alexa 647-A)

**0.19%**

**0.04%**

**0.02%**

IL-21 (Alexa 647-A)

IL-21 (Alexa 647-A)

IL-21 (Alexa 647-A)

**0.02%**

**0.03%**

**0.04%**

IL-21 (Alexa 647-A)

IL-21 (Alexa 647-A)

IL-21 (Alexa 647-A)

**0%**

**0.05%**

**0.01%**

IL-21 (Alexa 647-A)

IL-21 (Alexa 647-A)

IL-21 (Alexa 647-A)

**0.03%**

**0.04%**

**0.05%**

IL-21 (Alexa 647-A)

IL-21 (Alexa 647-A)

IL-21 (Alexa 647-A)

**0.05%**

**0.03%**

**0.03%**

IL-21 (Alexa 647-A)

IL-21 (Alexa 647-A)

IL-21 (Alexa 647-A)

V-III-SS-29

CD3 (BUV395-A)

V-III-SS-28

CD3 (BUV395-A)

V-III-SS-27

CD3 (BUV395-A)

V-III-SS-25

CD3 (BUV395-A)

V-III-SS-24

CD3 (BUV395-A)

V-III-SS-23

CD3 (BUV395-A)

V-III-SS-22

CD3 (BUV395-A)

V-III-SS-21

CD3 (BUV395-A)

V-III-SS-20

CD3 (BUV395-A)

V-III-SS-16

CD3 (BUV395-A)

V-III-SS-15

CD3 (BUV395-A)

V-III-SS-14

CD3 (BUV395-A)

V-III-SS-13

CD3 (BUV395-A)

V-III-SS-12

CD3 (BUV395-A)

V-III-SS-10

CD3 (BUV395-A)

V-III-SS-9

CD3 (BUV395-A)

V-III-SS-8

CD3 (BUV395-A)

V-III-SS-5

CD3 (BUV395-A)

V-III-SS-4

CD3 (BUV395-A)

V-III-SS-3

CD3 (BUV395-A)

V-III-SS-2

CD3 (BUV395-A)

V-III-SS-1

CD3 (BUV395-A)

CD3 (BUV395-A)

CD3 (BUV395-A)

CD3 (BUV395-A)

CD3 (BUV395-A)

CD3 (BUV395-A)

CD3 (BUV395-A)

CD3 (BUV395-A)

CD3 (BUV395-A)

CD3 (BUV395-A)

CD3 (BUV395-A)

CD3 (BUV395-A)

CD3 (BUV395-A)

CD3 (BUV395-A)

CD3 (BUV395-A)

CD3 (BUV395-A)

CD3 (BUV395-A)

CD3 (BUV395-A)

CD3 (BUV395-A)

CD3 (BUV395-A)

CD3 (BUV395-A)

CD3 (BUV395-A)

CD3 (BUV395-A)

CD3 (BUV395-A)

CD3 (BUV395-A)

CD3 (BUV395-A)

CD3 (BUV395-A)

CD3 (BUV395-A)

CD3 (BUV395-A)

CD3 (BUV395-A)

CD3 (BUV395-A)

CD3 (BUV395-A)

CD3 (BUV395-A)

CD3 (BUV395-A)

CD3 (BUV395-A)

CD3 (BUV395-A)

CD3 (BUV395-A)

CD3 (BUV395-A)

CD3 (BUV395-A)

CD3 (BUV395-A)

CD3 (BUV395-A)

CD3 (BUV395-A)

CD3 (BUV395-A)

CD3 (BUV395-A)

CD3 (BUV395-A)

CD3 (BUV395-A)

CD3 (BUV395-A)

CD3 (BUV395-A)

CD3 (BUV395-A)

CD3 (BUV395-A)

CD3 (BUV395-A)

CD3 (BUV395-A)

CD3 (BUV395-A)

CD3 (BUV395-A)

CD3 (BUV395-A)

CD3 (BUV395-A)

CD3 (BUV395-A)

CD3 (BUV395-A)

CD3 (BUV395-A)

CD3 (BUV395-A)

CD3 (BUV395-A)

CD3 (BUV395-A)

CD3 (BUV395-A)

CD3 (BUV395-A)

CD3 (BUV395-A)

CD3 (BUV395-A)

CD3 (BUV395-A)

CD3 (BUV395-A)

CD3 (BUV395-A)

CD3 (BUV395-A)

CD3 (BUV395-A)

CD3 (BUV395-A)

CD3 (BUV395-A)

CD3 (BUV395-A)

CD3 (BUV395-A)

CD3 (BUV395-A)

CD3 (BUV395-A)

CD3 (BUV395-A)

CD3 (BUV395-A)

CD3 (BUV395-A)

CD3 (BUV395-A)

CD3 (BUV395-A)

CD3 (BUV395-A)

CD3 (BUV395-A)

CD3 (BUV395-A)

CD3 (BUV395-A)

CD3 (BUV395-A)

CD3 (BUV395-A)

CD3 (BUV395-A)

CD3 (BUV395-A)

CD3 (BUV395-A)

CD3 (BUV395-A)

CD3 (BUV395-A)

CD3 (BUV395-A)

CD3 (BUV395-A)

CD3 (BUV395-A)

CD3 (BUV395-A)

CD3 (BUV395-A)

CD3 (BUV395-A)

CD3 (BUV395-A)

CD3 (BUV395-A)

CD3 (BUV395-A)

CD3 (BUV395-A)

CD3 (BUV395-A)

CD3 (BUV395-A)

CD3 (BUV395-A)

CD3 (BUV395-A)

CD3 (BUV395-A)

CD3 (BUV395-A)

CD3 (BUV395-A)

CD3 (BUV395-A)

CD3 (BUV395-A)

CD3 (BUV395-A)

CD3 (BUV395-A)

CD3 (BUV395-A)

**Supplementary Figure 6:** NS

S-pool

Baseline D14 post vac1 D14 post vac2 D14 post vac3 EOS Baseline D14 post vac1 D14 post vac2 D14 post vac3 EOS


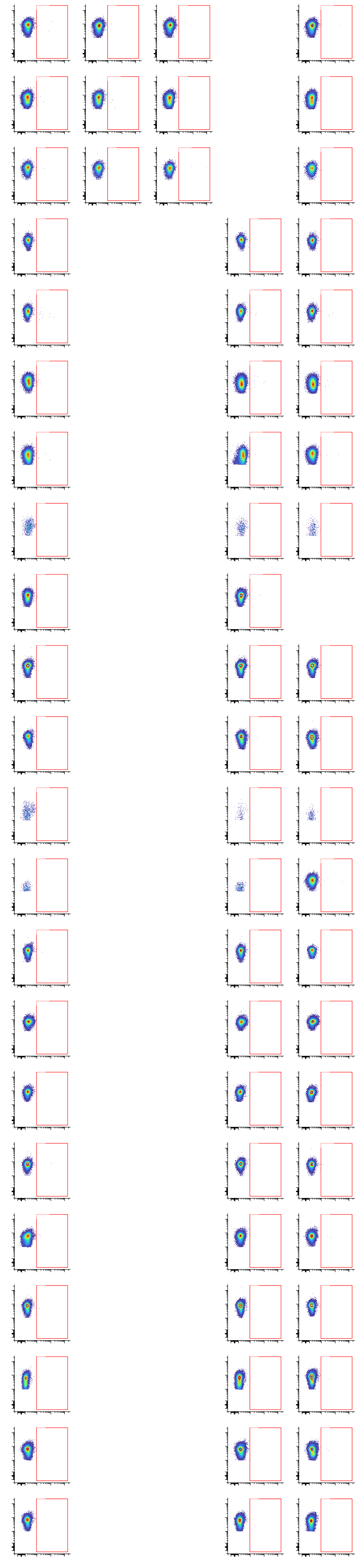


**0.01%**

**0%**

**0%**

**0.01%**

IFNg (FITC-A)

IFNg (FITC-A)

IFNg (FITC-A)

IFNg (FITC-A)

**0.01%**

**0.01%**

**0%**

**0%**

IFNg (FITC-A)

IFNg (FITC-A)

IFNg (FITC-A)

IFNg (FITC-A)

**0%**

**0%**

**0.01%**

**0.01%**

IFNg (FITC-A)

IFNg (FITC-A)

IFNg (FITC-A)

IFNg (FITC-A)

**0%**

**0%**

**0%**

IFNg (FITC-A)

IFNg (FITC-A)

IFNg (FITC-A)

**0.05%**

**0.02%**

**0.02%**

IFNg (FITC-A)

IFNg (FITC-A)

IFNg (FITC-A)

**0.03%**

**0.02%**

**0.01%**

IFNg (FITC-A)

IFNg (FITC-A)

IFNg (FITC-A)

**0.02%**

**0.02%**

**0.01%**

IFNg (FITC-A)

IFNg (FITC-A)

IFNg (FITC-A)

**0%**

**0%**

**0%**

IFNg (FITC-A)

IFNg (FITC-A)

IFNg (FITC-A)

**0%**

**0.01%**

IFNg (FITC-A)

IFNg (FITC-A)

**0%**

**0%**

**0%**

IFNg (FITC-A)

IFNg (FITC-A)

IFNg (FITC-A)

**0%**

**0%**

**0%**

IFNg (FITC-A)

IFNg (FITC-A)

IFNg (FITC-A)

**0.07%**

**0%**

**0%**

IFNg (FITC-A)

IFNg (FITC-A)

IFNg (FITC-A)

**0%**

**0%**

**0.01%**

IFNg (FITC-A)

IFNg (FITC-A)

IFNg (FITC-A)

**0.01%**

**0%**

**0%**

IFNg (FITC-A)

IFNg (FITC-A)

IFNg (FITC-A)

**0.01%**

**0%**

**0%**

IFNg (FITC-A)

IFNg (FITC-A)

IFNg (FITC-A)

**0%**

**0%**

**0%**

IFNg (FITC-A)

IFNg (FITC-A)

IFNg (FITC-A)

**0%**

**0%**

**0.01%**

IFNg (FITC-A)

IFNg (FITC-A)

IFNg (FITC-A)

**0.01%**

**0%**

**0%**

IFNg (FITC-A)

IFNg (FITC-A)

IFNg (FITC-A)

**0%**

**0%**

**0%**

IFNg (FITC-A)

IFNg (FITC-A)

IFNg (FITC-A)

**0%**

**0%**

**0%**

IFNg (FITC-A)

IFNg (FITC-A)

IFNg (FITC-A)

**0%**

**0%**

**0%**

IFNg (FITC-A)

IFNg (FITC-A)

IFNg (FITC-A)

**0%**

**0%**

**0%**

IFNg (FITC-A)

IFNg (FITC-A)

IFNg (FITC-A)


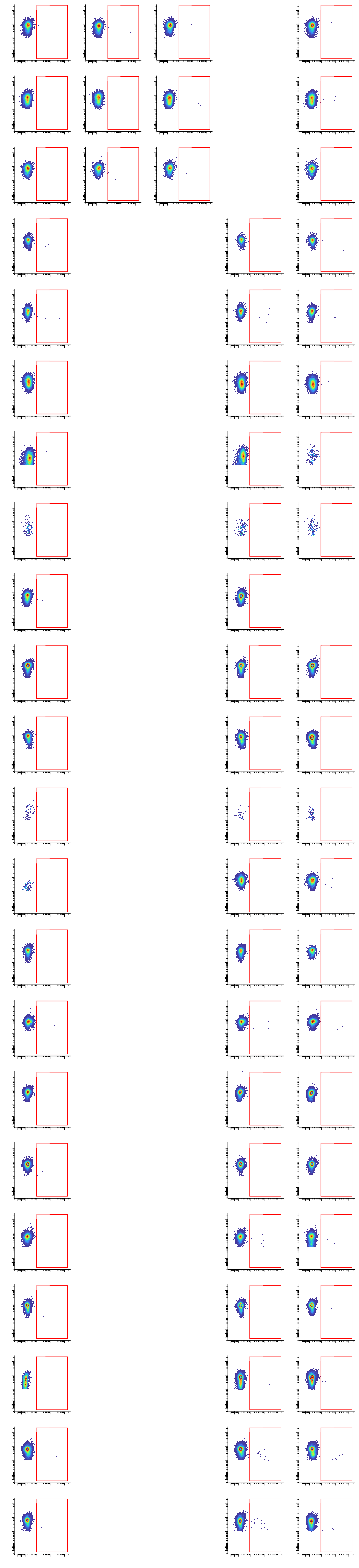


**0.01%**

**0.02%**

**0.01%**

**0.01%**

IFNg (FITC-A)

IFNg (FITC-A)

IFNg (FITC-A)

IFNg (FITC-A)

**0%**

**0.02%**

**0.01%**

**0.01%**

IFNg (FITC-A)

IFNg (FITC-A)

IFNg (FITC-A)

IFNg (FITC-A)

**0%**

**0.02%**

**0.06%**

**0.03%**

IFNg (FITC-A)

IFNg (FITC-A)

IFNg (FITC-A)

IFNg (FITC-A)

**0.02%**

**0.04%**

**0.06%**

IFNg (FITC-A)

IFNg (FITC-A)

IFNg (FITC-A)

**0.11%**

**0.13%**

**0.06%**

IFNg (FITC-A)

IFNg (FITC-A)

IFNg (FITC-A)

**0.01%**

**0.01%**

**0.01%**

IFNg (FITC-A)

IFNg (FITC-A)

IFNg (FITC-A)

**0.03%**

**0.03%**

**0%**

IFNg (FITC-A)

IFNg (FITC-A)

IFNg (FITC-A)

**0.11%**

**0.08%**

**0.1%**

IFNg (FITC-A)

IFNg (FITC-A)

IFNg (FITC-A)

**0.01%**

**0.02%**

IFNg (FITC-A)

IFNg (FITC-A)

**0%**

**0%**

**0%**

IFNg (FITC-A)

IFNg (FITC-A)

IFNg (FITC-A)

**0%**

**0.01%**

**0%**

IFNg (FITC-A)

IFNg (FITC-A)

IFNg (FITC-A)

**0.74%**

**0.3%**

**0%**

IFNg (FITC-A)

IFNg (FITC-A)

IFNg (FITC-A)

**0.09%**

**0.06%**

**0.02%**

IFNg (FITC-A)

IFNg (FITC-A)

IFNg (FITC-A)

**0.01%**

**0.01%**

**0.01%**

IFNg (FITC-A)

IFNg (FITC-A)

IFNg (FITC-A)

**0.1%**

**0.05%**

**0.03%**

IFNg (FITC-A)

IFNg (FITC-A)

IFNg (FITC-A)

**0.01%**

**0.01%**

**0%**

IFNg (FITC-A)

IFNg (FITC-A)

IFNg (FITC-A)

**0.02%**

**0.01%**

**0.03%**

IFNg (FITC-A)

IFNg (FITC-A)

IFNg (FITC-A)

**0.02%**

**0.07%**

**0.04%**

IFNg (FITC-A)

IFNg (FITC-A)

IFNg (FITC-A)

**0.01%**

**0.02%**

**0.01%**

IFNg (FITC-A)

IFNg (FITC-A)

IFNg (FITC-A)

**0%**

**0.01%**

**0.01%**

IFNg (FITC-A)

IFNg (FITC-A)

IFNg (FITC-A)

**0.02%**

**0.15%**

**0.08%**

IFNg (FITC-A)

IFNg (FITC-A)

IFNg (FITC-A)

**0.01%**

**0.14%**

**0.04%**

IFNg (FITC-A)

IFNg (FITC-A)

IFNg (FITC-A)

V-III-SS-29

CD3 (BUV395-A)

V-III-SS-28

CD3 (BUV395-A)

V-III-SS-27

CD3 (BUV395-A)

V-III-SS-25

CD3 (BUV395-A)

V-III-SS-24

CD3 (BUV395-A)

V-III-SS-23

CD3 (BUV395-A)

V-III-SS-22

CD3 (BUV395-A)

V-III-SS-21

CD3 (BUV395-A)

V-III-SS-20

CD3 (BUV395-A)

V-III-SS-16

CD3 (BUV395-A)

V-III-SS-15

CD3 (BUV395-A)

V-III-SS-14

CD3 (BUV395-A)

V-III-SS-13

CD3 (BUV395-A)

V-III-SS-12

CD3 (BUV395-A)

V-III-SS-10

CD3 (BUV395-A)

V-III-SS-9

CD3 (BUV395-A)

V-III-SS-8

CD3 (BUV395-A)

V-III-SS-5

CD3 (BUV395-A)

V-III-SS-4

CD3 (BUV395-A)

V-III-SS-3

CD3 (BUV395-A)

V-III-SS-2

CD3 (BUV395-A)

V-III-SS-1

CD3 (BUV395-A)

CD3 (BUV395-A)

CD3 (BUV395-A)

CD3 (BUV395-A)

CD3 (BUV395-A)

CD3 (BUV395-A)

CD3 (BUV395-A)

CD3 (BUV395-A)

CD3 (BUV395-A)

CD3 (BUV395-A)

CD3 (BUV395-A)

CD3 (BUV395-A)

CD3 (BUV395-A)

CD3 (BUV395-A)

CD3 (BUV395-A)

CD3 (BUV395-A)

CD3 (BUV395-A)

CD3 (BUV395-A)

CD3 (BUV395-A)

CD3 (BUV395-A)

CD3 (BUV395-A)

CD3 (BUV395-A)

CD3 (BUV395-A)

CD3 (BUV395-A)

CD3 (BUV395-A)

CD3 (BUV395-A)

CD3 (BUV395-A)

CD3 (BUV395-A)

CD3 (BUV395-A)

CD3 (BUV395-A)

CD3 (BUV395-A)

CD3 (BUV395-A)

CD3 (BUV395-A)

CD3 (BUV395-A)

CD3 (BUV395-A)

CD3 (BUV395-A)

CD3 (BUV395-A)

CD3 (BUV395-A)

CD3 (BUV395-A)

CD3 (BUV395-A)

CD3 (BUV395-A)

CD3 (BUV395-A)

CD3 (BUV395-A)

CD3 (BUV395-A)

CD3 (BUV395-A)

CD3 (BUV395-A)

CD3 (BUV395-A)

CD3 (BUV395-A)

CD3 (BUV395-A)

CD3 (BUV395-A)

CD3 (BUV395-A)

CD3 (BUV395-A)

CD3 (BUV395-A)

CD3 (BUV395-A)

CD3 (BUV395-A)

CD3 (BUV395-A)

CD3 (BUV395-A)

CD3 (BUV395-A)

CD3 (BUV395-A)

CD3 (BUV395-A)

CD3 (BUV395-A)

CD3 (BUV395-A)

CD3 (BUV395-A)

CD3 (BUV395-A)

CD3 (BUV395-A)

CD3 (BUV395-A)

CD3 (BUV395-A)

CD3 (BUV395-A)

CD3 (BUV395-A)

CD3 (BUV395-A)

CD3 (BUV395-A)

CD3 (BUV395-A)

CD3 (BUV395-A)

CD3 (BUV395-A)

CD3 (BUV395-A)

CD3 (BUV395-A)

CD3 (BUV395-A)

CD3 (BUV395-A)

CD3 (BUV395-A)

CD3 (BUV395-A)

CD3 (BUV395-A)

CD3 (BUV395-A)

CD3 (BUV395-A)

CD3 (BUV395-A)

CD3 (BUV395-A)

CD3 (BUV395-A)

CD3 (BUV395-A)

CD3 (BUV395-A)

CD3 (BUV395-A)

CD3 (BUV395-A)

CD3 (BUV395-A)

CD3 (BUV395-A)

CD3 (BUV395-A)

CD3 (BUV395-A)

CD3 (BUV395-A)

CD3 (BUV395-A)

CD3 (BUV395-A)

CD3 (BUV395-A)

CD3 (BUV395-A)

CD3 (BUV395-A)

CD3 (BUV395-A)

CD3 (BUV395-A)

CD3 (BUV395-A)

CD3 (BUV395-A)

CD3 (BUV395-A)

CD3 (BUV395-A)

CD3 (BUV395-A)

CD3 (BUV395-A)

CD3 (BUV395-A)

CD3 (BUV395-A)

CD3 (BUV395-A)

CD3 (BUV395-A)

CD3 (BUV395-A)

CD3 (BUV395-A)

CD3 (BUV395-A)
