# Supplementary material for: Development of a personalized digital biomarker of vaccine-associated reactogenicity using wearable sensors and digital twin technology
Source: Commun Med (Lond). 2025 Apr 13;5:115. doi: 10.1038/s43856-025-00840-8 (PMC11994808; doi:10.1038/s43856-025-00840-8)
Supplement: Supplementary file 3 — Description of Additional Supplementary File [file 43856_2025_840_MOESM3_ESM.pdf]

Description Of Additional Supplementary File

File name: Supplementary Data
Description: Supplementary Data for Manuscript Figures

File name: Supplementary Data 1
Supplementary data for manuscript figure 1

File name: Supplementary Data 2
Supplementary data for manuscript figure 2

File name: Supplementary Data 3
Supplementary data for manuscript figure 3

File name: Supplementary Data 4
Supplementary data for manuscript figure 4

File name: Supplementary Data 5
Supplementary data for manuscript figure 5
