# Supplementary material for: Development of a personalized digital biomarker of vaccine-associated reactogenicity using wearable sensors and digital twin technology
Source: Commun Med (Lond). 2025 Apr 13;5:115. doi: 10.1038/s43856-025-00840-8 (PMC11994808; doi:10.1038/s43856-025-00840-8)
Supplement: Supplementary file 4 — Supplementary Data [file 43856_2025_840_MOESM4_ESM.docx]

Supplementary Data for Manuscript Figures:

**Development of a personalized digital biomarker of vaccine-associated reactogenicity using wearable sensors and digital twin technology**

**Supplementary Data 1:**

1. Heart rate data
2. Heart rate variability (HRV) data
3. Respiratory rate data
4. Skin temperature data
5. Trailing activity data

**Supplementary Data 2**:

1. Individual variability in individual data types

**Supplementary Data 3:**

1. All participants heatmap data
2. Pfizer/BioNTech participants heatmap data
3. Moderna-only participants heatmap data

**Supplementary Data 4:**

1. MCIR histogram data
2. MCIR curve fit data

**Supplementary Data 5:**

1. Data for individual changes in individual MCIR AUC and geometric mean concentration (GMC) changes in antibody levels from baseline.
2. Curve fit data for MCIR AUC and GMC change.
3. Data for individual changes in individual MCIR AUC and interleukin-21 (IL-21+) expressing CD4+ cells
4. Curve fit data for MCIR AUC and interleukin-21 (IL-21+) expressing CD4+ cells
5. Data for individual changes in individual MCIR AUC and interferon-gamma (IFN-γ+) expressing CD8+ cells
6. Curve fit data for MCIR AUC and interferon-gamma (IFN-γ+) expressing CD8+ cells
