# Supplementary material for: Development of a personalized digital biomarker of vaccine-associated reactogenicity using wearable sensors and digital twin technology
Source: Commun Med (Lond). 2025 Apr 13;5:115. doi: 10.1038/s43856-025-00840-8 (PMC11994808; doi:10.1038/s43856-025-00840-8)
Supplement: Supplementary file 10 — REPORTING SUMMARY [file 43856_2025_840_MOESM10_ESM.pdf]

nature portfolio

Corresponding author(s): Steven R. Steinhubl

Last updated by author(s): Dec 10, 2024

Reporting Summary

Nature Portfolio wishes to improve the reproducibility of the work that we publish. This form provides structure for consistency and transparency in reporting. For further information on Nature Portfolio policies, see our Editorial Policies and the Editorial Policy Checklist.

Statistics

For all statistical analyses, confirm that the following items are present in the figure legend, table legend, main text, or Methods section.

n/a	Confirmed	
☐	☒	The exact sample size (*n*) for each experimental group/condition, given as a discrete number and unit of measurement
☒	☐	A statement on whether measurements were taken from distinct samples or whether the same sample was measured repeatedly
☐	☒	The statistical test(s) used AND whether they are one- or two-sided<br>*Only common tests should be described solely by name; describe more complex techniques in the Methods section.*
☐	☒	A description of all covariates tested
☐	☒	A description of any assumptions or corrections, such as tests of normality and adjustment for multiple comparisons
☐	☒	A full description of the statistical parameters including central tendency (e.g. means) or other basic estimates (e.g. regression coefficient) AND variation (e.g. standard deviation) or associated estimates of uncertainty (e.g. confidence intervals)
☐	☒	For null hypothesis testing, the test statistic (e.g. *F*, *t*, *r*) with confidence intervals, effect sizes, degrees of freedom and *P* value noted<br>*Give P values as exact values whenever suitable.*
☒	☐	For Bayesian analysis, information on the choice of priors and Markov chain Monte Carlo settings
☒	☐	For hierarchical and complex designs, identification of the appropriate level for tests and full reporting of outcomes
☒	☐	Estimates of effect sizes (e.g. Cohen's *d*, Pearson's *r*), indicating how they were calculated

Our web collection on statistics for biologists contains articles on many of the points above.

Software and code

Policy information about availability of computer code

Data collection	Data were collected on the physIQ (now Prolaio) propitiatory platform.
Data analysis	Python 3.8 was utilized on Google Compute Virtual Machines running the Linux operating system to generate analytics from wearable sensor data, including physiological features and Multivariate Change Index (MCI) results. Statistical analyses and chart generation were performed using The MathWorks MATLAB software, specifically versions R2017 and R2023, on Windows 10 systems.

For manuscripts utilizing custom algorithms or software that are central to the research but not yet described in published literature, software must be made available to editors and reviewers. We strongly encourage code deposition in a community repository (e.g. GitHub). See the Nature Portfolio guidelines for submitting code & software for further information.

Data

Policy information about availability of data

All manuscripts must include a data availability statement. This statement should provide the following information, where applicable:
- Accession codes, unique identifiers, or web links for publicly available datasets
- A description of any restrictions on data availability
- For clinical datasets or third party data, please ensure that the statement adheres to our policy

The data is the property of the sponsor and not publicly available for sharing.

Human research participants

Policy information about studies involving human research participants and Sex and Gender in Research.

Reporting on sex and gender	Participants were asked to self report their sex at the time of enrollment. At no other time in the study were sex or gender addressed.
Population characteristics	A total of 107 individuals consented to participate and included 137 vaccine doses. After excluding individuals with insufficient baseline or post-vaccine data, 88 participants were included in this analysis with a total of 104 vaccine doses. Forty-two (47.7%) were female and the mean age (+SD) for the analyzed population was 37.9 (+13.9) years with a range of 19 to 69 years. Eleven people (12.5%) self-reported prior COVID infection.
Recruitment	Participants were recruited through one of two study protocols. The majority of participants were enrolled through the Vaccine Induced Inflammation Investigation (VIII) study protocol. The second protocol was the Continuous Physiologic Monitoring for Immune Response via Wearable Sensor Data (COmMON SENS) study. For the VIII study, individuals who were already voluntarily planning to receive a vaccine against COVID-19 were recruited from the general population primarily via email outreach disseminated by employees of the study sponsor, physIQ (Chicago, IL, USA, but since purchased by Prolaio, Scottsdale, AZ, USA). Employees were encouraged to further disseminate recruitment information to their family and friends. In the COmMON SENS study, Purdue University students, staff, and faculty were recruited via advertising to the general university population, affiliates, and friends. Again, only individuals who were voluntarily planning to receive a COVID-19 vaccine were recruited.
Ethics oversight	Sterling IRB (ID# 8842-SRSteinhubl)

Note that full information on the approval of the study protocol must also be provided in the manuscript.

Field-specific reporting

Please select the one below that is the best fit for your research. If you are not sure, read the appropriate sections before making your selection.

- [x] Life sciences
- [ ] Behavioural & social sciences
- [ ] Ecological, evolutionary & environmental sciences

For a reference copy of the document with all sections, see nature.com/documents/nr-reporting-summary-flat.pdf

Life sciences study design

All studies must disclose on these points even when the disclosure is negative.

Sample size	As an exploratory study, a convenience sample of ~100 individuals was the initial goal.
Data exclusions	All data were included in analyses. When some data were excluded from specific analyses the reasoning is explained in the test.
Replication	*Describe the measures taken to verify the reproducibility of the experimental findings. If all attempts at replication were successful, confirm this OR if there are any findings that were not replicated or cannot be reproduced, note this and describe why.*
Randomization	*Describe how samples/organisms/participants were allocated into experimental groups. If allocation was not random, describe how covariates were controlled OR if this is not relevant to your study, explain why.*
Blinding	*Describe whether the investigators were blinded to group allocation during data collection and/or analysis. If blinding was not possible, describe why OR explain why blinding was not relevant to your study.*

Reporting for specific materials, systems and methods

We require information from authors about some types of materials, experimental systems and methods used in many studies. Here, indicate whether each material, system or method listed is relevant to your study. If you are not sure if a list item applies to your research, read the appropriate section before selecting a response.

Materials & experimental systems

n/a	Involved in the study	
☒	☐	Antibodies
☒	☐	Eukaryotic cell lines
☒	☐	Palaeontology and archaeology
☒	☐	Animals and other organisms
☐	☒	Clinical data
☒	☐	Dual use research of concern

Methods

n/a	Involved in the study	
☒	☐	ChIP-seq
☒	☐	Flow cytometry
☒	☐	MRI-based neuroimaging

Clinical data

Policy information about clinical studies

All manuscripts should comply with the ICMJE guidelines for publication of clinical research and a completed CONSORT checklist must be included with all submissions.

Clinical trial registration	clinicaltrials.gov NCT05237024
Study protocol	Propietary data of the sponsor
Data collection	Data was primarily collected via wearable sensors, A small substudy also had blood draws at CellCarta (Montreal, Qc, CA),
Outcomes	This was an exploratory study to develop a digital biomarker. The goal and priamry outcome was to explore the relationship of that digital biomalrker with self-reported post-vaccine symptoms. A secondary outcome was the relationship between the biomarker and immunogenicity data.
